# Independent short- and long-term dependencies in perception

**DOI:** 10.1167/jov.23.5.12

**Published:** 2023-05-15

**Authors:** Toni P. Saarela, Saija M. Niemi, Maria Olkkonen

**Affiliations:** 1 Department of Psychology and Logopedics, Faculty of Medicine, University of Helsinki, Haartmaninkatu 3, 00014 University of Helsinki, Helsinki, Finland

**Keywords:** serial depedence, central-tendency bias, perceptual bias, color appearance, psychophysics

## Abstract

Perception is biased by stimulus history. Both long-term effects such as the central-tendency bias (CTB) and short-term effects such as serial dependence (SD) have been described, but research into the two has remained largely separate. The sources of these effects, however, are highly correlated in stimulus statistics, which can result in a misinterpretation of experimental data. We compared CTB and SD in the perception of color and line length. Observers judged the relative hue or length of consecutive stimuli in a delayed-matching task. Two interstimulus intervals were used to investigate whether elapsed time or the number of stimulus occurrences was more important for SD. We estimated biases by fitting psychometric functions to the data split based on the history features, and we also fit generalized linear mixed models with either CTB, SD, or both included as regressors. We found biases to both recent stimulus history and the cumulative average of stimulus values for both color and line length judgments. The strength and pattern of each of the biases depended on whether all sources of bias were included in the analysis. Within the range of interstimulus intervals tested, the number of intervening stimuli was more important than elapsed time for SD. We conclude that both SD and CTB independently affect perceptual judgments, and that one effect is not an artifact caused by the other. Failing to consider both effects in data analysis can give an erroneous picture of the phenomenon under study.

## Introduction

It has been known for more than one hundred years that perception is biased by stimulus history: Both long-term effects whereby perception is biased by the distribution of stimuli encountered during an experiment, and short-term effects that depend on the stimulus sequence, have been described over a century ago (Fernberger, 1920; Hollingworth, 1910). Since then, numerous studies have been published on both the long-term “central tendency” effects (e.g., Ashourian & Loewenstein, 2011; Huttenlocher, Hedges, & Vevea, 2000; Jazayeri & Shadlen, 2010; Olkkonen, McCarthy, & Allred, 2014) and shorter-term “sequential” effects (e.g., Cicchini, Mikellidou, & Burr, 2017; Fischer & Whitney, 2014; Fründ, Wichmann, & Macke, 2014). These two research traditions, however, have been almost exclusively separate, and it is currently not known how short-term dependencies relate to long-term ones. This is problematic because, as we discuss elsewhere in this article, one type of effect can disguise as the other if the analysis fails to take both into account.

In a psychophysical experiment, immediately preceding stimuli bias judgments of the current one (Fernberger, 1920). Such biases have been called either “sequential effects” (Holland & Lockhead, 1968; Jesteadt, Luce, & Green, 1977; Parducci, 1964), “recency effects” (Raviv, Ahissar, & Loewenstein, 2012), or “serial dependencies” (SD) (Fischer & Whitney, 2014), and they have been found for a variety of stimulus dimensions, including orientation (Cicchini et al., 2017; Fischer & Whitney, 2014; Fritsche, Mostert, & de Lange, 2017; Pascucci et al., 2019), numerosity (Corbett, Fischer, & Whitney, 2011; Fornaciai & Park, 2018b), color (Barbosa & Compte, 2020), motion direction (Czoschke, Fischer, Beitner, Kaiser, & Bledowski, 2019), spatial position (Bliss, Sun, & D'Esposito, 2017), heading direction (Xu, Sun, Zhang, & Li, 2022), facial identity (Liberman, Fischer, & Whitney, 2014), facial attractiveness (Van der Burg, Rhodes, & Alais, 2019; Xia, Leib, & Whitney, 2016), loudness (Ward & Lockhead, 1970; Holland & Lockhead, 1968), and pitch (Raviv et al., 2012). The effect can be either attractive or repulsive and its magnitude depends on the difference in stimulus values and the time elapsed between them. The immediately preceding stimulus typically exerts an attractive perceptual bias: perception of the current stimulus is drawn towards the value of the preceding stimulus. This bias has been proposed to stabilize perception across time (e.g., Czoschke et al., 2019; Fischer et al., 2020; Fischer & Whitney, 2014) and to decrease errors in perception (Cicchini, Mikellidou, & Burr, 2018).

Stimulus history also affects perception on a much longer timescale: the distribution of stimulus values encountered during the course of an experiment exerts an attractive perceptual bias (Hollingworth, 1910), often called a central-tendency bias (CTB). The effect is seen in delayed-matching tasks, where the range of perceived stimulus values is compressed, that is, perceived stimulus values are drawn towards the center of the stimulus distribution. One suggestion for the origin of this effect is a Bayesian-type inference where sensory inputs are combined with prior expectations about stimulus prevalence (Ashourian & Loewenstein, 2011; Jazayeri & Shadlen, 2010). CTB is seen on several stimulus dimensions, including size (Hollingworth, 1910), shape (Huttenlocher et al., 2000), hue and lightness (Olkkonen et al., 2014, Olkkonen, Saarela, & Allred, 2016), line length (Ashourian & Loewenstein, 2011), numerosity (Xiang, Graeber, Enke, & Gershman, 2021), and interval duration (Jazayeri & Shadlen, 2010).

Although both SD and CTB are caused by stimulus history, they have almost exclusively been studied separately, and the commonly used analysis methods are constrained to reveal only the effect under study. This is remarkable, considering that in experiments focusing on SD, there is also a longer term stimulus history that might affect perceptual processing and in experiments focusing on CTB, conversely, the trials immediately preceding the current one might have biasing effects. The interpretation of experimental results, thus, might depend on the theoretical framework of the study.

**Figure 1.**
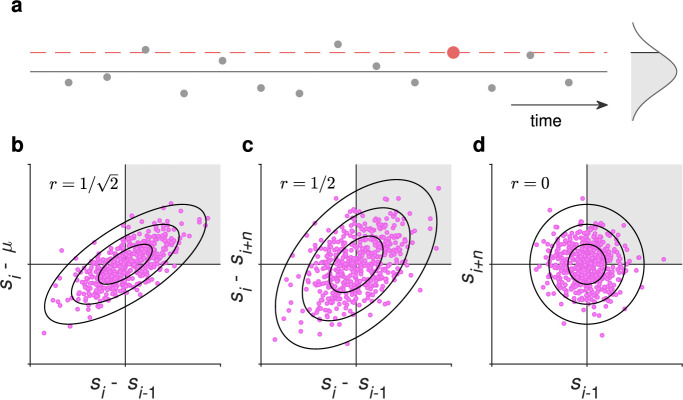
(a) When stimulus values (gray symbols) are drawn from a distribution at random on each trial and a given stimulus value (the large red symbol) is above the mean (black horizontal line), there is a higher probability that the current stimulus value is also higher than the stimulus value on a randomly chosen trial. (b) The difference between the current stimulus (*s*_*i*_) and the previous one (*s*_*i* − 1_) is correlated with the difference between the current stimulus and the mean (µ) with a correlation coefficient of $1/\sqrt{2}$. The region where both are positive is highlighted in gray. (c) The differences from the current stimulus to stimuli that are −1 and *n* trials removed from the current one are also correlated, with a correlation coefficient of 1/2, although the stimulus values themselves are uncorrelated (d).

This situation becomes even more problematic as the immediate and long-term stimulus histories are often correlated. Imagine that a stimulus value is drawn randomly from a distribution on each trial in an experiment. The cumulative mean of the stimuli already presented quickly tends to the distribution mean. When the mean is higher than the value of the current stimulus, the value of the previous stimulus is also more likely to be higher than the current one. When the mean is lower, the previous stimulus value is more likely to be lower as well (Figure 1a). That is, the difference between the current stimulus and the mean most likely has the same sign as the difference between the current stimulus and the one immediately preceding it. Failing to consider these alternative sources of bias when inferring the effects of stimulus history on perception from behavioral data might lead to misattribution of those effects. We are not the first to point this out (see, e.g., Sailor & Antoine, 2005; Tong & Dubé, 2022b), but this fact has not permeated the analysis methods used in the literature.

Figure 1b shows the correlation of the differences between the current stimulus and the one preceding it and between the current stimulus and the mean in an experiment where stimuli are drawn at random from a distribution. The correlation coefficient is $1/\sqrt{2}$ (the distribution shown in 1a is Gaussian, but the correlation coefficient does not depend on the distribution; see Appendix A). Similarly, even though the stimulus values on different trials (say, the previous trial *s*_*i* − 1_ and the trial *n* places removed from the current trial, *s*_*i* + *n*_) are independent as shown in Figure 1d, their differences to the current stimulus *s*_*i*_ are correlated, with a correlation coefficient of 1/2; this point is illustrated in Figure 1c. This is also why the data from a SD experiment can show spurious correlations, such as an apparent effect of the next trial on the current one, or a serial effect even after shuffling the stimulus order before analysis (Pascucci et al., 2019).

These correlations can lead to false conclusions about the source of perceptual bias if all potential sources of bias are not considered in the analysis. We designed an experiment and analyses to estimate the relative contributions of short-term (SD) and long-term (CTB) stimulus history on visual perception on two different stimulus dimensions: hue and line length. Because a sequential choice bias can also affect observer’s responses (Abrahamyan, Silva, Dakin, Carandini, & Gardner, 2016; Bosch, Fritsche, Ehinger, & de Lange, 2020; Senders & Sowards, 1952), we considered the effect of the preceding response in the analysis. We used a fixed stimulus timing where the observer’s response did not introduce any delays in the presentation, and two different interstimulus intervals (ISIs) to study whether the absolute time passed or the number of stimulus presentations drives SD effects. We found notable biases in the perception of hue and line length that are correlated with perceptual noise, with higher noise making perception more susceptible to bias. Judgments were also biased by the preceding response. We show that the perception of hue and the perception of line length are affected by both short-term and long-term stimulus history and describe the time course of these stimulus history–dependent effects.

## General methods

### Observers

Twelve naïve observers participated in the first experiment on hue. All observers were female and their average age was 21.2 years (range, 19–26 years). Observers had normal or corrected visual acuity and normal color vision as assessed with the Ishihara color plates. Twelve new naïve observers participated in the second experiment on line length. Eight observers were female, three were male and one was nonbinary, and their average age was 24.1 years (range, 19–31 years). All observers had normal or corrected visual acuity.

Before participating, observers signed informed consent. As compensation for their time, observers received either a gift card worth 20 euros or course credit.

### Apparatus

The experiments were run on a HP Z230 Desktop PC and implemented in Matlab (R2016b) with PsychToolbox (Brainard, 1997; Kleiner, Brainard, & Pelli, 2007; Pelli, 1997). Stimuli were displayed on a 23-inch ViewPixx monitor (1,920 × 1,080 px, 100 Hz) with 10-bit intensity resolution per color channel. The maximum luminance of the screen was 250 cd/m^2^. The viewing distance was held constant at 100 cm with a chin rest, and the experiment was completed in a darkened room. Observers gave their responses with a handheld RESPONSEPixx response box.

### Stimuli

#### Hue experiment

The stimuli were colored, circular disks displayed in the center of the screen. The disk diameter was 3° of visual angle.

Stimulus colors were defined in CIELAB color space. The white point of the monitor that was used as the reference for CIELAB had a luminance of 250 cd/m^2^ and was metameric to D65. The stimuli differed only in hue: all stimuli had a lightness (L*) of 55 and a chroma (C) of 40. Five equally spaced reference hue angles were used: 156°, 168°, 180°, 192°, and 204°. The hue of the comparison stimulus was controlled using a staircase procedure.

The stimuli were displayed on a noisy checkerboard background. The average color of the background was L* = 50, a* = 0, and b* = 0 in CIELAB space. The noise was uniformly distributed and varied ±5 units in each dimension. The background was updated during each intertrial interval.

#### Line length experiment

The stimuli were white, horizontal line segments (luminance 250 cd/m^2^) presented against a uniform gray (125 cd/m^2^) background. All line segments had a width of 0.2° of visual angle. The location was randomized around the center of the screen with a maximum distance from the center of 0.5°. Two ranges of reference stimuli were used. The short range was from 1.5° to 3.1°, and the long range was from 3.1° to 6.2°. Thus, the longest reference stimulus in the short range and the shortest length in the long range were the same. Reference stimulus lengths were spaced equally on a logarithmic scale.

### Procedure

Two different inter-stimulus intervals (ISIs) were used in separate blocks of trials. The short ISI block had an ISI of 500 ms and took approximately 15 minutes to complete. The long ISI block had an ISI of 1,500 ms and took approximately 30 minutes to complete. Each block consisted of 400 trials with the order of reference stimuli randomized. Observers had an opportunity to take a break at least every 100 trials. Observers completed one short block and one long block during a 1-hour session. Each observer participated in two sessions, so each block was completed twice. In the line length experiment, one session had the short range and the other had the long range. The order of blocks was counterbalanced between observers.

**Figure 2.**
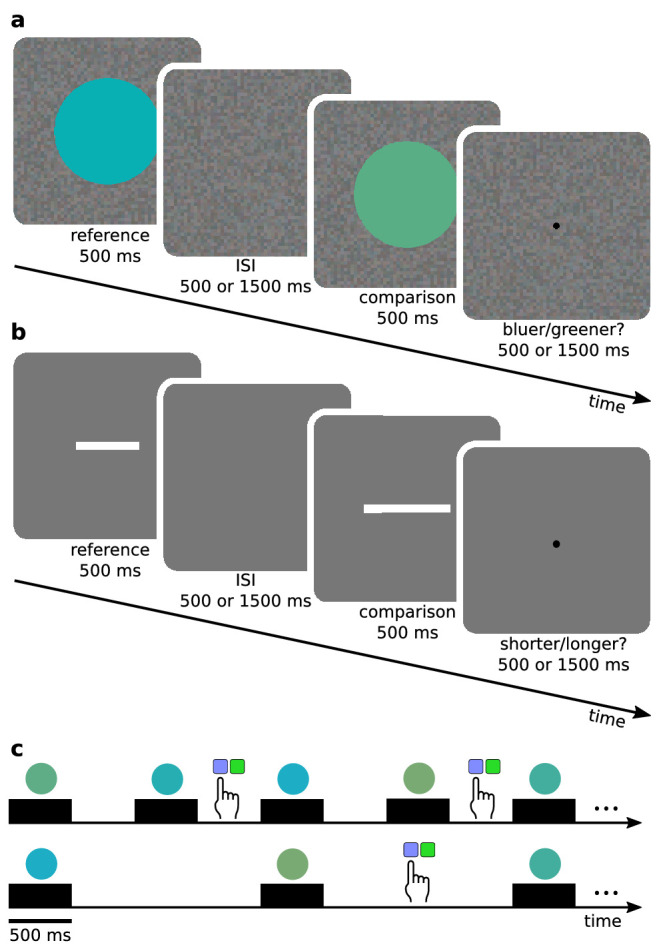
Experimental procedure. (a) For each trial in the hue experiment, the observer compared the hue of the reference and comparison stimuli, and answered whether the comparison appeared bluer or greener than the reference. (b) In the line length experiment, the observer compared the lengths of the reference and comparison line segments and replied whether the comparison seemed to be shorter or longer than the reference. (c) Two different timings were used. Stimulus duration was always 500 ms, but the ISI was either 500 ms (short ISI) or 1,500 ms (long ISI). The observer gave their answer after the comparison stimulus, and the experiment did not pause to wait for responses.

In each trial (Figure 2), a reference stimulus was presented for 500 ms, followed by a background noise texture for the duration of the ISI, and then a comparison stimulus for 500 ms. After the comparison stimulus, a black fixation dot was presented for the duration of the ISI, and observers were instructed to respond during the black dot. In the hue experiment, the observers’ task was to respond if the comparison stimulus was bluer or greener than the reference stimulus. In the line length experiment, observers responded if the comparison stimulus was longer or shorter than the reference stimulus. Observers gave their responses by pressing a corresponding button on a response box. To keep stimulus timing constant, the experiment program did not wait for observers’ responses, but continued to the next trial after the ISI had passed. Observers were instructed to avoid missing responses and calmly continue from the next trial if they missed one, for example, owing to a lapse in concentration. If the observer did not respond or responded late five trials in a row, the program forced the observer to take an extra break. Both experiments had a total of 19,200 trials. In the hue experiment, there were 127 missed trials, or 0.66% of all trials. In the line experiment, there were 299 missed trials, or 1.55%.

The value of the comparison stimulus was controlled by a staircase procedure. There were four interleaved staircases for each reference. Two of them had an initial value smaller than the target (a “1-up, 1-down” or 1-1 staircase and a 2-1 staircase), and two had a higher value (1-1 and 1-2 staircases).

### Analysis

#### Psychometric functions

We fit psychometric functions (PFs) (cumulative Gaussian) to the proportion of “bluer” (hue experiment) or “longer” (line length experiment) responses with the maximum likelihood method. The mean parameter was taken as the point of subjective equality and the standard deviation as the discrimination threshold and an estimate of internal noise. Bias in the perceived value was calculated as the difference between point of subjective equality and the true stimulus value.

To evaluate the effect of stimulus and response history on judgments of the current stimulus, we split the data based on the history features and fit new PFs to the split data. This process included splitting the data in two based on whether the current reference value was smaller or larger than one of the preceding stimuli or the cumulative mean, as well as based on the previous response. We also estimated the bias caused by the cumulative mean and preceding trials while controlling for other history features. For example, when estimating the bias caused by the cumulative mean, we chose for the analysis only those trials where the reference stimulus value was identical with, or very close to, the preceding stimulus.

To visualize how the bias depends on the difference in stimulus values, we binned the data based on the difference from the current reference to the mean and to preceding stimuli, and then fit PFs to the data in each bin.

#### Regression

The relative contributions of the history effects were quantified by fitting a generalized linear mixed model (GLMM) with a probit link function to the response data, with observer as a random factor. The GLMM was fit separately to the short ISI and long ISI data by maximum likelihood. We included the current stimuli (*s*_*i*, cmp_ and *s*_*i*, ref_), the cumulative mean of all stimuli (${\bar{s}}_{i}$), and average stimulus values from eight preceding trials (*t*_*i* − *j*_, *j* = 1…8) in the model. Our regressors thus suffer from collinearity as discussed in the Introduction and visualized in Figure 1, but partial collinearity does not violate the assumptions of GLM and does not bias the estimated coefficients (Kutner, Nachtsheim, Neter, & Li, 2005). To account for a possible sequential choice effect, the preceding response (*r*_*i* − 1_) was included as well. We used the average of the reference and comparison from preceding trials because their values are highly correlated; the comparison values were selected around the reference value. Our model observer combines information from the cumulative mean and preceding trials with the reference stimulus, and compares this information against the comparison stimulus. We thus included as regressors the differences between the comparison and reference, as well as differences between the reference and each of the stimulus history features. The effect of preceding stimuli, however, is not linear with the difference in stimulus values, but is well-described by a derivative of Gaussian (DoG) function (Fischer & Whitney, 2014; also confirmed by our own analysis). Therefore, the final regressors were the values of a DoG function with this difference as input. The linear model on trial *i* is

$$\begin{array}{ccc} \;\;\;\;\;\;\;\;\;\;\;\;y_{i}^{*} & \;= & \beta_{0}+\beta_{1}\Delta s_{i}+\beta_{2}\Delta {\bar{s}}_{i}\\ & & +\sum_{j=1}^{8}\beta_{i+2}g\left(\Delta t_{i-j}\right)+\beta_{11}r_{i-1}+ɛ, \end{array}$$

where Δ*s*_*i*_ = *s*_*i*, cmp_ − *s*_*i*, ref_, $\Delta {\bar{s}}_{i}=s_{i,\text{ref}}-{\bar{s}}_{i}$, Δ*t*_*i* − *j*_ = *s*_*i*, ref_ − *t*_*i* − *j*_, *g* is the DoG function, and ε represents noise. *t*_*i* − *j*_ is the average stimulus value on trial *j* before the current trial. The DoG function has one parameter for the width. We varied the width parameter and always refit the GLMM to find the best-fitting value. This is a nonstandard fitting procedure in the sense that we fit the GLMM several times while varying the DoG width parameter. The alternative would be to fit a nonlinear mixed model, but our simpler approach is able to answer the main question about the independence of central tendency bias and SD. In addition to this full model, we also fit a model without the serial effects, and a version without the mean effect. Confidence intervals for the parameters were estimated by resampling the data 1,000 times and fitting the model to the sampled data.

For a more meaningful comparison of the weights given to different history effects, as well as comparison between the two experiments, we scaled the coefficients β for the stimulus terms so that the weight for the comparison stimulus was 1, making the total weight given to the reference stimulus and the history features also 1. This scaling can be thought of as transforming the comparison coefficient to stimulus units, and the inverse of the scaling factor gives the standard deviation in stimulus units, which should correspond well with the standard deviation from the PF fits. The weights reported in Figure 8 are valid for small stimulus differences where the DoG function is nearly linear, estimated using a linear approximation of the DoG function near zero. A more complete description of the GLM and the computation of these weights is given in Appendix B.

**Figure 3.**
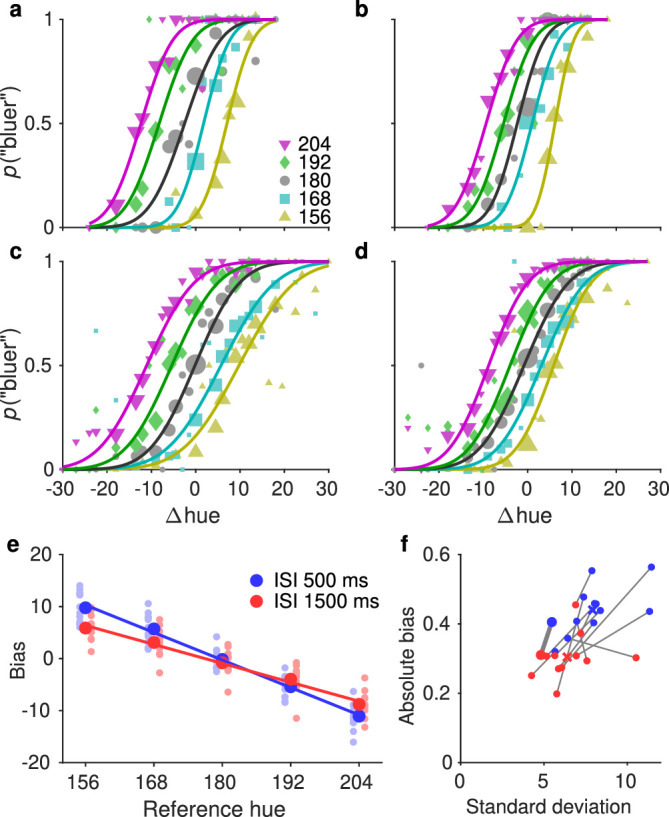
Bias in the hue experiment. (a, b) PFs for an example observer in the short ISI (a) and long ISI (b) conditions. The probability of judging the comparison stimulus to be bluer than the reference is plotted against hue difference between the comparison and reference. If there was no bias, the PFs would overlap and all cross the 0.5 probability at Δhue of 0. (c, d) Same as a and b, pooled data from all observers. (e) Bias extracted from the PFs, plotted against the reference hue. Small symbols are for individual observers, large symbols for pooled data. Bias in perceived hue was toward the central hues (positive for reference values lower than average, negative for reference values higher than average). The straight lines show a fit of a model with a single noise parameter and where the bias is linearly related to the reference value. (f) Bias strength (negative of the slope of the linear fit, see (e)) plotted against the internal noise estimate for individual observers. Lines connect data points for the two ISI conditions within observers. The crosses show averages, and the slightly larger symbols and thicker line highlight the example observer from a and b. Higher noise is associated with a larger bias.

**Figure 4.**
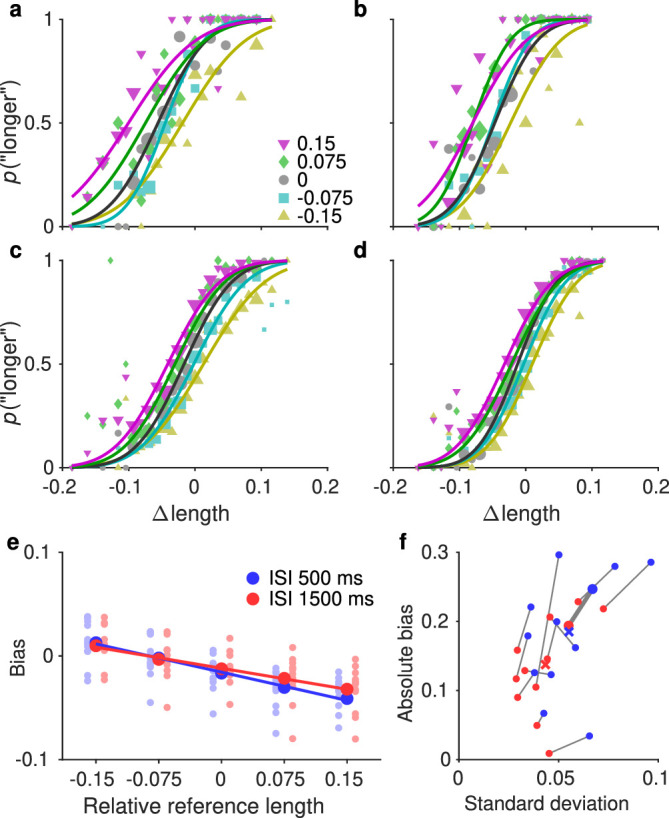
Bias in the line length experiment. (**a**, **b**) PFs for an example observer in the short ISI (**a**) and long ISI (**b**) conditions. (**c**, **d**) Same as a and b, pooled data from all observers. (**e**) Bias extracted from the PFs, plotted against the reference line length. The x-axis values are differences to the mean reference value, not absolute log lengths, because there were two length ranges and the data are pooled here. (**f**) Bias strength plotted against the internal noise estimate for individual observers. See caption to Figure 3 for more details.

**Figure 5.**
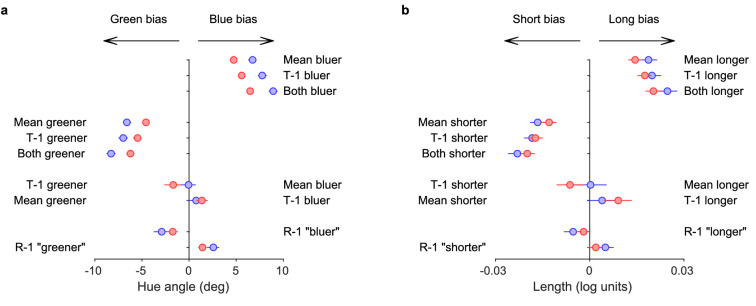
Bias in perceived reference value when the data were split based on the history features. T-1 indicates the stimulus value on the preceding trial, R-1 indicates the preceding response. (a) Hue experiment. The values on the x-axis show the bias toward green or blue. Different lines show the biases when the data were split in different ways. The text on each line indicates the split. Lines 7 and 8 show the bias when the data were split so that the differences to the cumulative mean and the preceding trial had opposite sign. (b) Line length experiment, format similar to a. The mean of a PF fit to all data was subtracted from the biases shown here to eliminate any constant bias unrelated to history features. Data were pooled across observers. Error bars (when visible from behind the symbol) show bootstrapped 95% confidence intervals. Colors: blue=short ISI, red=long ISI.

**Figure 6.**
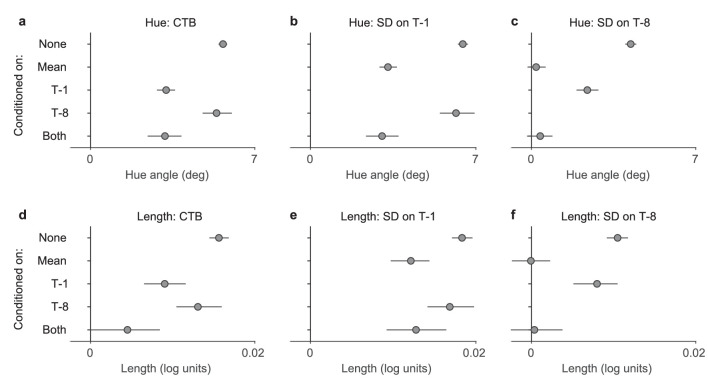
CTB and SD conditioned on stimulus history features. Data were pooled across the two ISIs, and positive values indicate attractive biases. (a) CTB in the hue experiment, conditioned on the serial effects from trials T-1, T-8, or both being zero. (b) SD on the preceding trial T-1, conditioned on the effect of cumulative mean or the trial T-8 being zero. (c) SD on the preceding trial T-8, conditioned on the effect of cumulative mean or the trial T-1 being zero. (d–f) Same as above, line length experiment. Error bars show bootstrapped 95% confidence intervals.

**Figure 7.**
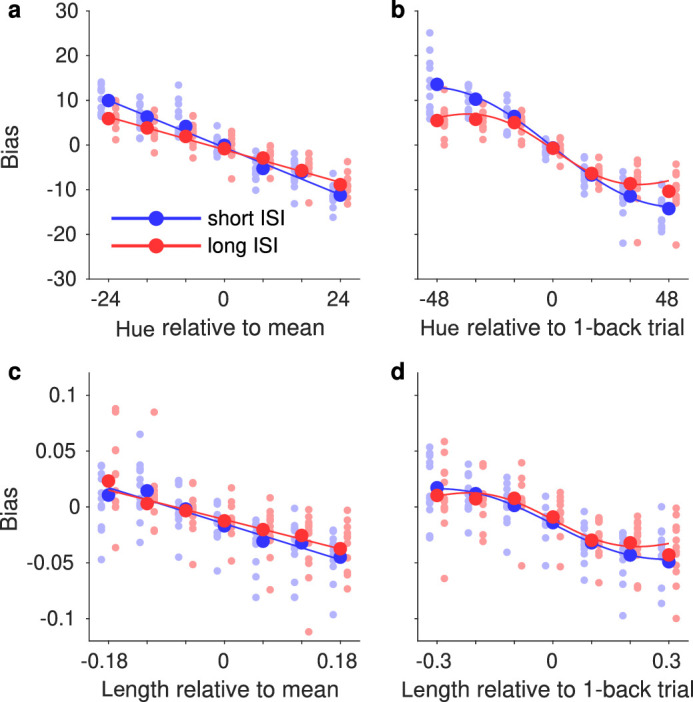
Bias as a function of the difference between the reference stimulus and history features. (a) The bias in perceived hue as a function of the difference from the reference stimulus to the cumulative mean is linear over the whole range. (b) The bias in perceived hue as a function of the difference to the preceding trial is nonlinear, as observed previously. Similar pattern of results holds for biases in perceived line length (c, d).

**Figure 8.**
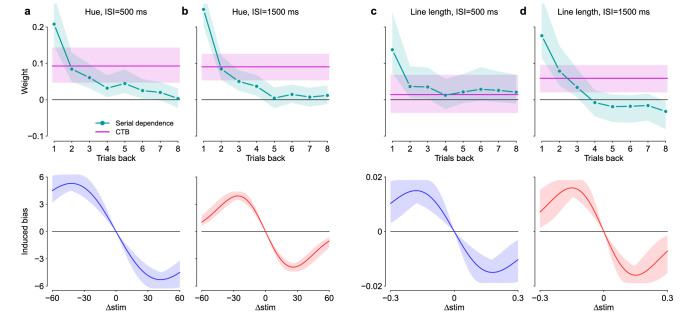
Time-course of SD and nonlinearity of SD. The top panel shows the strength of SD from the past eight trials. CTB weight shown in magenta for comparison. Shaded areas show bootstrapped 95% confidence intervals. The lower panel shows the best-fitting DoGs function that describes how SD depends on the difference in stimulus values between the current and past trials. (a) Hue, ISI 500 ms. (b) Hue, ISI 1,500 ms. (c) Line length, ISI 500 ms. (d) Line length, ISI 1,500 ms.

## Results

Perceived hue was biased in a way that systematically depended on the reference value: Small reference values had a positive bias, and large reference values had a negative bias. Figure 3 shows PFs for each reference from an example observer (a, b) as well as averaged over observers (c, d) for the short ISI (a, c) and long ISI (b, d) conditions. PFs are plotted as a function of the difference between the comparison and reference stimuli. If there were no bias, all PFs would overlap and have a mean of zero. Instead, the biased means can be seen clearly in both the individual and average data. Figure 3e plots the bias as a function of the reference value, showing the typical CTB effect. Larger biases were associated with higher noise, as shown in Figure 3f, which plots the absolute bias against the standard deviation of the PF fit.

Somewhat surprisingly, biases were larger and noise was higher in the short ISI condition. We expected a long ISI to increase noise and biases, because the stimulus had to be kept in memory for longer before the comparison. The short ISI condition was, however, noisier—possibly because the stimuli were presented at a constant rate without pausing for responses, which made the short ISI condition quite challenging. Nevertheless, the association between higher noise and larger bias was evident for the average data as well as on an individual level.

Similar trends held in the line length experiment, as seen in Figure 4. The difference in bias strength was not quite as pronounced as with hue, but higher noise was still clearly associated with a stronger bias.

To see how closely the bias scales with noise, we compared the biases in the short- and long-ISI conditions using *t*-tests. We compared both the raw biases reported in Figures 3f and 4f, and these same biases normalized by the noise (standard deviation of the PFs). There was a significant difference between the two ISI conditions with the unnormalized biases, hue: *t*(11) = 6.3, *p* < 0.001; line length: *t*(11) = 2.9, *p* = 0.013, but the difference diminished when the biases were normalized, hue: *t*(11) = 2.2, *p* = 0.051; line length: *t*(11) = 0.8, *p* = 0.416.

The analysis so far shows that the perception of different reference stimuli was biased to different extents, but as explained in the Introduction, it does not reveal the origin of those biases. We next analyzed the data not in terms of the reference values themselves (as is common in the central tendency literature), but in terms of the difference between the reference and the cumulative mean, as well as the reference and stimulus values on preceding trials and the previous response. These analyses were done on the data pooled over all observers.

Figure 5 shows the biases when the data were split based on these differences for a) hue and b) line length. The biases owing to stimulus history were attractive: if, for example, in the hue experiment the cumulative mean or the preceding trial (T-1) was bluer than the reference, the perception of the reference was biased towards blue. If both were bluer than the reference, the bias was even stronger. Similar attractive biases occurred toward green (5a), as well as long and short values (5b). If the cumulative mean and the preceding trial were in opposite directions from the reference, the bias was greatly reduced or eliminated, indicating that these effects cancel each other to a large extent. Finally, the observers had a tendency to switch responses: a preceding “reference bluer” response, for example, led to a bias toward green on the next trial.

To further investigate the independence of the stimulus-dependent history effects, we estimated the perceptual bias owing to the cumulative mean (CTB), the preceding trial (T-1), and the trial T-8, with the condition that the other history effects were close to zero. For example, conditioning on the cumulative mean meant that we only included in the analysis those trials where the cumulative mean was within a small tolerance range from the current reference value. Figure 6 shows the results. Data were pooled across ISIs, and mirrored and pooled with respect to the history features so that positive values indicate attractive bias. In perceived hue (a–c), CTB as well as SD from trials 1 and 8 before the current one were significant when not conditioned on the other features. When conditioning on T-1, T-8, or both, the CTB effect was still significant, although its magnitude was smaller when conditioning on T-1 (a). Similarly, the SD on T-1 was significant even after conditioning on the cumulative mean and T-8, with conditioning on the cumulative mean decreasing its magnitude (b). The SD on T-8, in contrast, disappeared when conditioned on the mean or both the mean and T-1, indicating that its apparent effect might be due to the correlations among the stimulus history features (c). Similar patterns again held for biases in perceived line length (d–f), with one exception: the CTB was diminished more when conditioned on both T-1 and T-8, with the 95% confidence interval just reaching zero (panel d; the confidence interval is also larger due to the smaller number of trials left when conditioning on two other features).

One notable observation from this analysis is that SD from trial T-8 survived conditioning on T-1, but not on the cumulative mean. This finding might at least partly be due to the fact that the correlation between the difference to the mean and to another trial is stronger (Figure 1b) than the correlations between differences to two different trials (Figure 1c). Note also that, for illustration, only two past trials (T-1 and T-8) were included in this analysis (and further conditioning would also have left very little data for PF fitting). The GLMM analysis below takes all trials from T-1 to T-8 into account.

Next, we binned the data based on the differences to the cumulative mean and the previous trial, and fit PFs to the data in each bin. The results are shown in Figure 7. The bias as a function of the difference to the cumulative mean was linear over the whole range, but the bias as a function of the difference to the preceding trial was clearly nonlinear and tapered off when the difference was large enough. This is consistent with previous reports that have found the same nonlinearity and suggested the effect size is well described by a DoG function (e.g., Fischer & Whitney, 2014). Note that the bias is plotted here as a function of the difference between the reference and the history features, so attractive biases are shown as positive for negative differences, and as negative for positive differences as is usual in the CTB literature. In some papers on SD, the reverse is true, because the differences are taken the other way.

We then investigated the origin of the biases by fitting a GLMM to the data, with observer as a random factor. Table 1 shows the results from the hue experiment. The first column shows the coefficients for a full model that includes both central tendency and SD effects. The second column shows a model with only central tendency and no serial effects. Finally, the third column shows a model with only serial effects. In the full model, the cumulative mean and several of the preceding trials had a significant effect on the responses. In addition, the preceding response also had an effect: there was a tendency for the observer to switch responses between trials, and this effect was consistent across experiments. When only the cumulative mean was included in the model, its effect was greatly overestimated: the coefficient is larger in the second column compared with the first column. This is due to the correlations among the different sources of bias explained in the Introduction: If serial effects are present but excluded from the analysis, they are absorbed by the estimated central tendency effect. Similarly, when only serial effects are included, their strength is also overestimated, although to a lesser extent. Further, when the mean was not included in the model, serial dependencies seemingly extended further into the past.

**Table 1.** Results from the GLMM for the hue experiment. Models were fit separately to the short and long ISI conditions. For both ISIs, three models were fit: A model including both central tendency and serial effects, a model including only central tendency effects, and a model including only serial effects. In all models, the previous response was also included. Δ*s* is the difference between the comparison and reference values on the current trial, $\Delta \bar{s}$ is the difference from the reference to the cumulative mean (source of CTB), Δ*t*_−*i*_ is the difference from the reference to the *i*th back trial (source of SD), and *r*_−1_ is the previous response, coded as −1 or 1.

	Short ISI			Long ISI		
	β, both	β, CTB	β, SD	β, both	β, CTB	β, SD
Intercept	0.076	0.086	0.074	0.182*	0.172*	0.177*
Δ*s*	0.133***	0.071***	0.131***	0.164***	0.106***	0.161***
$\Delta \bar{s}$	0.012***	0.056***		0.015***	0.046***	
Δ*t*_−1_	0.704***		0.773***	0.643***		0.708***
Δ*t*_−2_	0.285***		0.344***	0.219***		0.271***
Δ*t*_−3_	0.206***		0.259***	0.130***		0.177***
Δ*t*_−4_	0.108**		0.160***	0.095**		0.145***
Δ*t*_−5_	0.151***		0.207***	0.011		0.049
Δ*t*_−6_	0.085*		0.139***	0.038		0.087**
Δ*t*_−7_	0.068		0.115**	0.019		0.061*
Δ*t*_−8_	0.011		0.056	0.031		0.081**
*r*_−1_	−0.172***	−0.200***	−0.173***	−0.154***	−0.166***	−0.152***
LL	−4030.678	−4558.829	−4036.997	−3870.895	−4441.457	−3882.123
Akaike information criterion	8087.356	9127.657	8097.995	7767.789	8892.914	7788.246

In the long ISI condition, if only serial effects were included in the model, the effect disappeared in the fifth trial into the past, but became significant again in trials 6 to 8. These effects might be spurious, resulting from the exclusion of central tendency effects from the model. When both central tendency and serial effects were included, SD was significant until the fourth trial into the past, and not significant thereafter.

Table 2 shows the GLMM results for the line length experiment. Preceding trials again had a significant effect, but some differences also emerged compared with the hue experiment. First, the cumulative mean had a significant effect only with the long ISI; in the short ISI condition, the model without the central-tendency effect gave a slightly better fit than the full model as judged by the Akaike information criterion (the values are, however, very close to each other). Second, the serial effects did not extend as many trials back as with hue. The pattern was similar, however, in that the immediately preceding trial had the largest effect, and the effect quickly diminished.

**Table 2.** Results from the GLMM for the line length experiment. See Table 1 for details.

	Short ISI			Long ISI		
	β, both	β, CTB	β, SD	β, both	β, CTB	β, SD
Intercept	0.318***	0.311***	0.317***	0.314*	0.307**	0.313*
Δ*s*	18.022***	14.852***	18.005***	23.555***	20.001***	23.445***
$\Delta \bar{s}$	0.250	3.173***		1.388***	3.132***	
Δ*t*_−1_	0.270***		0.276***	0.377***		0.411***
Δ*t*_−2_	0.072*		0.078**	0.169***		0.209***
Δ*t*_−3_	0.069*		0.075**	0.073*		0.109***
Δ*t*_−4_	0.024		0.030	−0.017		0.012
Δ*t*_−5_	0.042		0.048	−0.041		−0.012
Δ*t*_−6_	0.056		0.062*	−0.039		−0.017
Δ*t*_−7_	0.051		0.056*	−0.034		−0.009
Δ*t*_−8_	0.041		0.046	−0.069*		−0.039
*r*_−1_	−0.077***	−0.090***	−0.077***	−0.037*	−0.056***	−0.037*
LL	−4237.832	−4605.013	−4238.007	−3921.164	−4297.167	−3926.205
Akaike information criterion	8501.664	9220.027	8500.013	7868.328	8604.334	7876.409

In the short ISI condition, although the central tendency effect was not significant, including it in the model nonetheless seemed to smooth out the variation in the coefficients for serial effects. When the central tendency effect was included, trials 1 through 3 into the past had a significant effect. When excluding it, effects from trials 6 and 7 became significant, although the effects from trials 4 and 5 remained not significant.

In the long ISI condition, attractive SD diminished to zero by three trials back and turned slightly negative (repulsive) for trials thereafter, reaching statistical significance at trial T-8. This is similar to the results of Fritsche, Spaak, and de Lange (2020), who found in a delayed orientation adjustment task an attractive bias for the most recent trial that turned repulsive over a few trials back.

The coefficients for different effects cannot be directly compared, because the effect of the preceding trials was weighted by a DoG function. We next converted the coefficients into weights that can be compared, taking the DoG function into account. This was done using a linear approximation to the DoG function and thus it only applies for small stimulus differences. Figure 8a–d show the weights computed from the coefficients. The estimated central-tendency effect is shown in magenta, and the time-course of SD for the past eight trials is shown in cyan.

Figures 8a, b show the time-course of SD for hue. The immediately preceding trial had by far the greatest effect, which then rapidly declined. SD extended further into the past with short ISI as measured in trials. This does not scale with absolute time: trials 1 to 4 back had about the same effect magnitude, although the lag in seconds was very different: 8 seconds versus 16 seconds for the short and long ISIs, respectively. The central-tendency effect had roughly the same magnitude with both short and long ISI. The serial effect from the one-back trial was larger than the estimated central-tendency effect, but starting from the third or fourth trial back, the serial effect was clearly weaker compared with central tendency.

Figures 8c, d show the time-course of SD for perception of line length. Compared to hue, SD was slightly weaker and had an effect over a shorter time scale. But, similar to hue, the immediately preceding trial had a large effect that then rapidly declined. SD was significant for up to three trials back for both ISI conditions, again showing that it was less dependent on elapsed time than on the number of intervening stimuli. The CTB was not significant with the short ISI, but became significant with long ISI.

Finally, the lower panels in Figure 8 show the estimated DoG function that best described how serial effects depend on the difference in stimulus values. The function has a single parameter that determines its width. For hue, the function was wider in the short ISI condition. For line length, the estimates were very similar for both ISI conditions. We also fit the GLMM without transforming the stimulus differences with the DoG function, and the results were qualitatively similar. But because the nonlinearity does exist, the magnitude of SD for small stimulus differences will be underestimated if it is not taken into account in the modeling.

## Discussion

We designed an experimental protocol and analysis to study the independent contributions of CTB and SD on perceptual judgments in a binary choice task. Because these factors are inherently correlated in stimulus sequences (Figure 1), they can be confused for one another if these correlations are not taken into account in the data analysis. Indeed, our results depended on whether we included all effects in the analysis or omitted one or the other effect. When controlling for each factor by either fitting PFs to split data or with a GLMM, we found significant and independent contributions of CTB and SD, and also found a significant sequential choice bias on both hue and line length judgments.

When analyzing the data separately for each reference, we found a robust bias toward the mean for both hue and line length. Smaller reference values were perceived as larger, and larger values smaller, with the magnitude of the bias correlating with noise estimated for individual observers. This finding is in line with previous results for hue (Olkkonen et al., 2014) and line length (Ashourian & Loewenstein, 2011). In these and other previous studies on CTB, however, short-term serial dependencies or response biases were not taken into account, so it is unclear how much of the bias in previous reports was in fact due to a bias toward the central tendency instead of a bias toward recent trials. To further investigate the sources of bias in our data, we estimated perceptual biases when the data were split based on the difference between the reference value and the history features. We found evidence for both CTB and SD from the preceding trial and showed that these effects can together cause an even larger bias or, in contrast, can cancel each other if their signs differ. This finding suggests that these two effects are both real and that one is not an artifact due to the correlations mentioned above. Similarly, controlling for the source of CTB weakened but did not eliminate SD from the preceding trial, and vice versa. SD from eight trials into the past was eliminated when controlling for CTB, and CTB was eliminated in the short ISI condition of the line length experiment when controlling for SD from trials 1 and 8 into the past. All these effects were seen in the GLMM results as well, as discussed below.

For hue, the CTB was approximately the same magnitude for the short and long ISIs based on the GLMMs, but for line length, there was a significant bias only for the long ISI when serial biases were accounted for. For both experiments and ISIs, we found strong attractive SD that quickly diminished and disappeared after four to six trials. For line length, the pattern of SD without CTB in the analysis was more spurious than with central tendency included. For hue, SD seemed to extend further into the past (up to eight trials for the long ISI) without a CTB term in the model. In general, the results of the GLMMs show that excluding some history features from the analysis can lead to an overestimation of other effects. Further, in the case of SD, excluding the central tendency effect from the model can lead to more spurious SD estimates as a function of time.

The preceding trial always caused the largest SD, which then diminished over several trials. When all factors were included in the GLMM, the bias for the one-back trial was also always larger than the CTB, which is in line with previous results (see Experiment 3 in Tong & Dubé, 2022b). The comparison of the falloff of SD across trials for the short and long ISIs revealed that it did not just depend on elapsed time. This finding was especially evident in line length judgments, where the bias clearly depended on the number of trials instead of elapsed time. For the long ISI condition, which is comparable with previous SD studies in terms of trial duration, the SD remained significant for 16 (hue) and 12 (line length) seconds, which is on the same order as in previous reports, although note that the elapsed time varies from trial to trial in studies that use adjustment tasks (e.g., Fischer & Whitney, 2014; Tong & Dubé, 2022b; Van der Burg et al., 2019). However, to our knowledge our work is the first systematic test of the relative importance of elapsed time and number of intervening stimuli for SD.

In visual perception, central tendency and serial biases have been traditionally characterized for achromatic stimuli, such as lines, two-dimensional shapes and gratings. Especially for serial effects, a commonly used task is magnitude estimation or adjustment. Employing a binary choice task, we previously found a CTB that correlated with observer uncertainty for hue (Olkkonen et al., 2014) and lightness (Olkkonen et al., 2016), but did not analyze the data for serial effects. Barbosa and Compte (2020) recently reported serial dependencies for hue in a re-analysis of color working memory data from several experiments. The present work is to our knowledge the first demonstration of SD for color in a binary choice task, and the first systematic characterization of independent central tendency and serial effects on any visual dimension.

Some previous work has considered both short-term and long-term biases in sequential judgments and found both (Chopin & Mamassian, 2012; DeCarlo & Cross, 1990; Gekas, McDermott, & Mamassian, 2019; Huang & Sekuler, 2010; Sailor & Antoine, 2005) or just the other (Duffy, Huttenlocher, Hedges, & Crawford, 2010; Tong & Dubé, 2022b). Chopin and Mamassian (2012) found correlations between previously seen stimuli and current responses with binocular rivalry and tilt aftereffect stimuli. Current responses correlated negatively with the most recent stimuli and positively with stimuli in a more remote window (see also Gekas et al., 2019). The repulsive bias to recent history is a rare exception among SD studies, but may be explained by the particularities in experimental procedure; repulsive biases have been reported with a short ISI either towards previous stimuli (Bliss et al., 2017) or the stimulus mean (Olkkonen et al., 2014) and Fritsche et al. (2017) found a repulsive effect towards the previous trial for 2AFC orientation judgments, akin to a tilt after-effect. Sailor and Antoine (2005) also found both short-term and long-term biases in a size adjustment task: size estimates were attracted both towards the mean size of all stimuli and towards the size of the previous stimulus. The two sources of bias, however, appear to have been tested in separate analyses, an approach which will not account for correlations between the two factors. Duffy et al. (2010) found line length estimates to be biased towards the cumulative mean but not towards recent trials, but Crawford's (2019) re-analysis of the data (in response to Duffy & Smith, 2018) showed a bias towards the cumulative mean as well as towards the average of the past 3 stimuli. Recently, Tong and Dubé (2022b) found both central tendency and serial biases in a spatial frequency estimation task, but explained both effects with one underlying process whereby past trials are weighted based on their reliability and integrated to produce the current percept. Although this fidelity-based weighting produces data that seemingly contains both central tendency and serial effects, the framework is agnostic about possible independence of these effects in experimental data. Based on our results, we conclude that these effects are separate and at least partly independent.

There is active debate in the SD literature whether the effect originates at the level of perception (e.g., Cicchini, Benedetto, & Burr, 2021; Cicchini et al., 2017; Fornaciai & Park, 2018a; Murai & Whitney, 2021; Rafiei, Hansmann-Roth, Whitney, Kristjánsson, & Chetverikov, 2021) or rather postperceptually (e.g., Bliss et al., 2017; Ceylan, Herzog, & Pascucci, 2021; Feigin, Baror, Bar, & Zaidel, 2021; Fritsche et al., 2017; Moon & Kwon, 2022). Bliss et al. (2017) showed by manipulating the delay in a spatial delayed response task that SD at the group level was repulsive for an immediate response and became increasingly attractive for delays up to six seconds, with substantial individual differences in the magnitudes of repulsive and attractive biases. This pattern led the authors to conclude that the effect is mnemonic rather than perceptual. Additionally, the authors showed with an intertrial interval manipulation that the effect did not just depend on the passage of time, in line with what we found. Manassi, Liberman, Kosovicheva, Zhang, and Whitney (2018) arrived at a different conclusion based on a similar position estimation paradigm based on their finding that SD did occur for an immediate response when the target contrast was low. Cicchini et al. (2021) used the surround tilt illusion to test whether SD was toward the perceived or physical orientation. By analyzing SD separately for neutral and illusory trials as a function of the type of preceding trial, they showed that SD incorporated perceptual orientation but acted on perception at a level before the illusion took place. Furthermore, Ceylan et al. (2021) found SD across different stimulus types, which is a strong indication that SD is not exclusively perceptual in origin, although it can act on perception, and some results from ensemble coding imply late temporal integration that occurs at the level of short-term memory (Tong & Dubé, 2022a; Zepp, Dubé, & Melcher, 2021). Our experiments were not designed to reveal the locus of SD, but we did find that both central tendency bias and SD behave similarly on the two different visual dimensions tested. This finding suggests either that the biases originate at a level beyond early processing of hue and length, or that similar mechanisms operate independently on these feature dimensions.

In the aggregate, our observers tended to alternate responses in all conditions. Most SD studies have reported repeating responses (e.g., Akaishi, Umeda, Nagase, & Sakai, 2014; Braun, Urai, & Donner, 2018; Senders & Sowards, 1952), although Fernberger (1920) found alternating responses. Zhang and Alais (2020) found that observers repeat previous choices, but that their motor responses are repulsed. Senders and Sowards (1952) found that subjects tend to repeat responses when they think they are discriminating small stimulus differences but the stimuli are in fact identical, reinforcing a point by Skinner (1942) that alternating responses can be an explicit strategy. We were primarily interested in CTB and SD, and we included the preceding response in the model only as a control so that it would not interfere with other bias estimates. We found both CTB and SD even when controlling for response bias, suggesting that they are separable from responses.

To conclude, sequential perceptual estimation in a binary choice task is biased independently towards the cumulative mean of the stimuli and to the past three to six trials. These biases are not reducible to a sequential choice bias. These results have implications to all studies employing stimulus sequences, but especially those aiming to characterize short- and long-term perceptual biases.

## Appendix A: Correlations between differences of stimulus values and the mean

Let *s*_*i*_ be independent and identically distributed stimulus value presented on trials *i* = 1…*n*, with a common mean µ and common variance σ^2^. The expected value of the difference between stimulus values on any two trials *i* and *j* is naturally 0, as is the difference between the stimulus value on trial *i* and the mean µ:

$$\begin{array}{ccc} E[s_{i}-s_{j}] & = & E\left[s_{i}\right]-E\left[s_{j}\right]=\mu -\mu =0\\ E[s_{i}-\mu ] & = & E[s_{i}]-\mu =\mu -\mu =0. \end{array}$$

The corresponding variances are Var[*s*_*i*_ − *s*_*j*_] = 2σ^2^ and Var[*s*_*i*_ − µ] = σ^2^.

Next, look the covariance between the differences from the stimulus on trial *i* to stimuli on trials *j* and *k*, and the covariance between the differences from the stimulus on trial *i* to the stimulus on trial *j* and to the mean µ. Because in both cases any shared variance must come from *s*_*i*_, it is also intuitively clear that the covariance must be σ^2^:

$$\begin{array}{c} \;\;\;\;\;\;\;\;\;\;\;\;\;\begin{array}{ccc} \mathrm{Cov}[\left(s_{i}-s_{j}\right),\left(s_{i}-s_{k}\right)] & \;= & E\left[\left(s_{i}-s_{j}\right)\left(s_{i}-s_{k}\right)\right]-E\left[s_{i}-s_{j}\right]E\left[s_{i}-s_{k}\right]\\ & \;= & E[s_{i}^{2}-s_{i}s_{k}-s_{i}s_{j}+s_{j}s_{k}]\\ & \;= & E\left[s_{i}^{2}\right]-E\left[s_{i}\right]E\left[s_{j}\right]-E\left[s_{i}\right]E\left[s_{k}\right]+E\left[s_{j}\right]E\left[s_{k}\right]\\ & \;= & E\left[s_{i}^{2}\right]-\mu^{2}\\ & \;= & \mathrm{Var}[s_{i}]\\ & \;= & \sigma^{2}\\ \mathrm{Cov}[\left(s_{i}-s_{j}\right),\left(s_{i}-\mu \right)] & \;= & E\left[\left(s_{i}-s_{j}\right)\left(s_{i}-\mu \right)\right]-E\left[s_{i}-s_{j}\right]E\left[s_{i}-\mu \right]\\ & \;= & E[s_{i}^{2}-s_{i}\mu -s_{i}s_{j}+s_{j}\mu ]\\ & \;= & E\left[s_{i}^{2}\right]-E\left[s_{i}\right]E\left[s_{j}\right]-\mu E\left[s_{i}\right]+\mu E\left[s_{j}\right]\\ & \;= & E\left[s_{i}^{2}\right]-\mu^{2}\\ & \;= & \mathrm{Var}[s_{i}]\\ & \;= & \sigma^{2}. \end{array} \end{array}$$

Finally, the correlations are

$$\begin{array}{c} \;\;\;\;\;\;\;\;\;\;\;\;\;\begin{array}{ccc} \mathrm{Cor}[\left(s_{i}-s_{j}\right),\left(s_{i}-s_{k}\right)] & \;= & \frac{\mathrm{Cov}[\left(s_{i}-s_{j}\right),\left(s_{i}-s_{k}\right)]}{\sqrt{2}\sigma \sqrt{2}\sigma }=\frac{\sigma^{2}}{2\sigma^{2}}=\frac{1}{2}\\ \mathrm{Cor}[\left(s_{i}-s_{j}\right),\left(s_{i}-\mu \right)] & \;= & \frac{\mathrm{Cov}[\left(s_{i}-s_{j}\right),\left(s_{i}-\mu \right)]}{\sqrt{2}\sigma \sigma }=\frac{\sigma^{2}}{\sqrt{2}\sigma^{2}}=\frac{1}{\sqrt{2}}. \end{array} \end{array}$$

## Appendix B: GLM and computation of stimulus weights

This section gives a more thorough explanation of the GLM and describes the calculation of the weights shown in Figure 8. Consider first using a GLM for fitting a PF to our data. The model for trial *i* is

⇒yi=β0+β1si,cmp-si,ref

⇒yi=Φ-1(p)

$$\begin{array}{c} \;\Rightarrow p=\Phi \left(\beta_{0}+\beta_{1}\left(s_{i,\text{cmp}}-s_{i,\text{ref}}\right)\right)\; \end{array}$$

⇒yi=Φ(si,cmp-si,ref)-μσ,

where *s*_*i*, cmp_ and *s*_*i*, ref_ are the comparison and reference stimuli, Φ( · ) is the standard normal distribution function, and *p* is the probability of a response (“bluer” or “longer” in our case). The overall bias in stimulus units is thus µ = −β_0_/β_1_ and the standard deviation of the PF is σ = 1/β_1_.

We modeled the effect of the history features on the processing of the reference stimulus. We included the cumulative mean of all stimuli (${\bar{s}}_{i}$), and average stimulus values from eight preceding trials (*t*_*i* − *j*_, *j* = 1…8), as well as the preceding response in the model (*r*_*i* − 1_). We first ignore the nonlinearity related to the effect of preceding trials. The observer combines information from the cumulative mean and preceding trials with the reference stimulus, and compares this against the comparison stimulus. The decision variable (or latent variable) on trial *i* is

$$\begin{array}{ccc} y_{i}^{*} & \;= & \alpha_{0}+\alpha_{1}s_{i,\text{cmp}}+\alpha_{2}s_{i,\text{ref}}+\alpha_{3}{\bar{s}}_{i}+\alpha_{4}t_{i-1}\\ & & +...+\alpha_{11}t_{i-8}+\alpha_{12}r_{i-1}+ɛ, \end{array}$$

where ε represents zero-mean Gaussian noise. Because the observer judges the comparison against the combination of the reference and the history features, this gives a constraint $\alpha_{1}=-\left(\alpha_{2}+\alpha_{3}+...+\alpha_{11}\right)\Rightarrow \sum_{i=1}^{11}\alpha_{i}=0$, so that the constant term α_0_ captures any bias not related to previous stimuli or responses and there is one fewer coefficient to fit. We thus use stimulus differences to the reference as regressors, and the model is

$$\begin{array}{c} \;\;\;\;\;\;\;\;\;\;\;\;\;y_{i}^{*}=\beta_{0}+\beta_{1}\Delta s_{i}+\beta_{2}\Delta {\bar{s}}_{i}+\sum_{j=1}^{8}\beta_{i+2}\Delta t_{i-j}+\beta_{11}r_{i-1}+ɛ, \end{array}$$

where Δ*s*_*i*_ = *s*_*i*, cmp_ − *s*_*i*, ref_, $\Delta {\bar{s}}_{i}=s_{i,\text{ref}}-{\bar{s}}_{i}$, Δ*t*_*i* − *j*_ = *s*_*i*, ref_ − *t*_*i* − *j*_. We can now rescale these coefficients to stimulus units by dividing by the coefficient for the comparison, β_1_. This is the same scaling as in the example of simple PF fitting above. This also means that the weights given to the reference and the stimulus history features sum to minus one. These are analogous to the weights that are reported for the history features in Figure 8 with a sign convention so that attractive bias is positive. The only difference in the model we used was the nonlinearity applied to the stimulus differences to previous trials. In accordance with previous literature, we used a derivative-of-Gaussian (DoG, scaled to 0–1) function to weight these differences. The weights for the history features in stimulus units cannot then be computed directly from the regression coefficients, but when the stimulus differences Δ*t* are small, we can use a linear approximation to the DoG function. That is, we can scale the coefficients by the absolute value of the second DoG evaluated at zero. For example, the weight for the cumulative mean $\bar{s}$ is

$$\begin{array}{c} w_{\bar{s}}=\frac{e^{0.5}}{\sigma_{\mathrm{DoG}}}\frac{\beta_{2}}{\beta_{1}}, \end{array}$$

where σ_DoG_ is the width parameter. The coefficients for preceding trials are scaled similarly. These weights are reported in Figure 8.
